# Cultural Biases and Clinical Variation

**DOI:** 10.3390/clinpract15120229

**Published:** 2025-12-05

**Authors:** Óscar Gasulla, Antonio Sarría-Santamera, Ferran A. Mazaira-Font, Cielo García-Montero, Oscar Fraile-Martinez, Diego Cantalapiedra, Manuel F. Carrillo-Rodríguez, Belen Gómez-Valcárcel, Miguel Á. Ortega, Melchor Álvarez-Mon, Angel Asúnsolo

**Affiliations:** 1 Hospital Universitari de Bellvitge, Universitat de Barcelona, L’Hospitalet de Llobregat, 08907 Barcelona, Spain; oscargasulla@gmail.com; 2 Department of Surgery, Medical and Social Sciences, Faculty of Medicine and Health Sciences, University of Alcalá, 28801 Alcala de Henares, Spain; manuel.carrillo@uah.es (M.F.C.-R.); belengomval@hotmail.com (B.G.-V.); 3 Department of Biomedical Sciences, Nazarbayev University School of Medicine, Astana 010000, Kazakhstan; antonio.sarria@nu.edu.kz; 4 Departament d’Econometria, Estadística i Economia Aplicada, Universitat de Barcelona, 08907 Barcelona, Spain; ferranmazaira@gmail.com; 5 Department of Medicine and Medical Specialities, Faculty of Medicine and Health Sciences, University of Alcalá, 28801 Alcala de Henares, Spain; cielo.gmontero@gmail.com (C.G.-M.); oscarfra.7@gmail.com (O.F.-M.); miguel.angel.ortega92@gmail.com (M.Á.O.); mademons@gmail.com (M.Á.-M.); 6 Ramón y Cajal Institute of Sanitary Research (IRYCIS), 28034 Madrid, Spain; 7 Faculty of Medicine, Universidad Europea of Madrid, 28670 Madrid, Spain; diego151099@gmail.com; 8 Networking Research Center on for Liver and Digestive Diseases (CIBEREHD), Immune System Diseases-Rheumatology and Internal Medicine Service, University Hospital Prince of Asturias, 28806 Alcala de Henares, Spain; 9 Department of Epidemiology and Biostatistics, Graduate School of Public Health and Health Policy, University of New York, New York, NY 10027, USA

**Keywords:** clinical biases, clinical variation, social stigma, healthcare quality

## Abstract

**Background**: This study investigates the possible role of cultural biases in clinical variation. **Methods**: An observational, analytical, and retrospective cohort design, using a Spanish primary care database, was used, on which a cross-sectional analysis was performed. Diseases were classified into the following: generic conditions with established protocols (control group), specialist-diagnosed diseases, women’s diseases, and psychiatric diseases. Diagnostic incidence was calculated across 25 geographical clusters, and variability was measured using the coefficient of variation (CV). To test systematic differences, multivariate linear regression models were adjusted for socio-demographics and healthcare system proxies. **Results**: The final sample for analysis consisted of 617,222 women aged 15 to 65, with a mean age of 43. The results did not reveal significant variability between generic and specialized diseases, but women’s and psychiatric diseases showed consistently higher CVs in all models (*p* < 0.05), indicating that approximately 20% to 30% of the clinical variation observed in these groups was unexplained. **Conclusions**: Although we cannot establish causality, these findings may suggest that cultural taboos and gender biases likely contribute to unjustified clinical variation, highlighting cultural bias as a plausible explanatory factor.

## 1. Introduction

Clinical variation describes the different spectrum of healthcare practices and services applied to different population groups, focusing on the overuse, underuse, different use, and waste of healthcare practices and services with varying outcomes, to ultimately find ways to improve healthcare practices. In other words, reducing clinical variation contributes to delivering the right care at the right costs. Many reasons for clinical variation have been found. Among warranted clinical variation causes, we find geographic variation, corresponding to clusters of populations with different demographic and lifestyle characteristics, or the difference in patient’s preferences in undergoing different treatments available [1]. The clinical variation that stems from geographic variation and patient preferences may influence patient outcomes and applicable patient care options, but is not the focus of this paper.

Several studies find different accessibility to medical resources, such as different diagnostic tests, treatments, or access to medical specialists, as the main reason for differences in observed unwarranted clinical variation. Such a phenomenon represents a loss in healthcare service quality for the patients and could be amended with the standardization of protocols and a different distribution of healthcare resources [2].

Yet, even in a population with no significant geographic variation and acceptable allocation of medical resources, there is a percentage of observable clinical variation that remains unexplained. Several studies have analyzed other possible factors (such as clinical complexity, lack of definitive scientific evidence, clinician characteristics, etc.) [2]. However, there is a lack of empirical studies that tackle the existence of non-rational factors so far, namely, cognitive biases.

Cognitive biases are non-verified hypotheses we use as cognitive shortcuts to aid decision-making. Cultural biases are a type of cognitive bias that are socially and culturally conditioned, that is, systematic errors shaped by cultural norms, values, or taboos [3]. Biases seem to be a ubiquitous phenomenon and do not correlate whatsoever with intelligence nor any other measure of cognitive ability [4].

In clinical practice, cognitive bias is common. Cognitive errors have been identified in all steps of the diagnostic process, including information gathering, association triggering, context formulation, processing, and verification. Evidence shows that 78.9% of diagnostic errors in primary care involve cognitive bias during the patient encounter [5].

Nowadays, almost everywhere on a worldwide basis, mental illnesses have a strong social stigma [6]. Even in most developed countries, such as European countries, they are one of the strongest stigmatizations [6,7,8,9,10,11]. Social stigma and beliefs about mental illness affect the definition of these disorders. They also affect patients’ readiness and willingness to seek and adhere to treatment [12] as well as doctors’ diagnoses [13,14,15].

Regarding women’s health, there is substantial evidence of gender bias in clinical practice [16,17]. Men are investigated and treated more extensively than women with the same severity of symptoms in a large variety of diseases, such as coronary artery disease [18,19,20], Parkinson’s disease [21], tuberculosis [22], irritable bowel syndrome [23], neck pain [24], and orthopedic surgery [25]. Research indicates that physicians are more likely to under-estimate female patients’ pain compared with males pain, to interpret men’s symptoms as organic and women’s as psychosocial [24], and to prescribe more painkillers for male patients and more psychotherapy for female patients [26].

In order to test to what extent cultural bias is a root cause of clinical variation, we compare diagnostic variation between four groups of illnesses, using a primary care database elaborated in Spain with 617,222 female patients. In summary, we are testing to what extent diseases whose diagnosis could be influenced by cultural biases (social norms and cultural taboos) present higher incidence variability than other diseases. To do so, we compare the variability of psychiatric and women’s diseases concerning generic diseases with established protocols. If there were no differences, no bias would be affecting the diagnosis. If differences existed, they could be explained by cultural biases or by other factors.

## 2. Patients and Methods

### 2.1. Study Design

The present study was an observational, analytical, and retrospective cohort design on which a cross-sectional analysis was performed. This study was carried out with a medical primary care database collected during the year 2017. The database consists of 617,222 women, aged from 15 to 65. The age caps were chosen to assure a representative sample of women’s diseases (which are more prevalent during reproductive ages) and psychiatric diseases.

### 2.2. Characteristics of Patients

Two types of information were provided for each patient: socio-demographical and medical. Socio-demographical data describes age, Spanish region of origin, income—which is classified in four groups (less than EUR 18,000 per year, between EUR 18,000 and 99,999 per year, and, finally, more than EUR 100,000 per year), and size of the municipality of origin, classified in five groups (less than 10,000 people, between 10,000 and 49,999, between 49,999 and 99,999, between 100,000 and 499,999, and more than 500,000 people). Medical data describes codified diseases for every patient with an opening and closing date (if it exists), visits registered at primary medical centers, and medication prescribed with an opening and closing date (if it exists).

Four groups of pathologies were created to conduct the study:

1. Generic diseases with established protocols (control group): Arterial hypertension, dyslipidemia, diabetes, obesity, asthma, ischemic cardiopathy, tobacco abuse, bronchitis, dementia, and heart failure met these conditions [27,28,29,30,31,32,33,34].
2. Diseases with established protocols diagnosed by specialists: In this group, we chose a set of 20 diseases managed by different medical specialists. We selected one or three diseases per specialty to have a representative wide sample among the diagnoses available in our database. This group consists of tuberculosis, leukemia, retinal detachment, macular degeneration, otosclerosis, auditory system cancer, cardiac valvopathy, cerebral stroke, pulmonary embolism, respiratory system cancer, rheumatoid arthritis, multiple sclerosis, Parkinson’s disease, epilepsy, psoriasis, thyroid cancer, renal cancer, and urinary bladder cancer.
3. Women’s diseases: We selected all diseases exclusive to women, with affectation of the breast or female reproductive system. Concretely, these were as follows: endometriosis, contraception interventions, feminine infertility, disability associated with gestation and menstruation, fibrocystic mastopathy, uterine fibromyoma, inflammatory pelvic disease, and vulvovaginitis.
4. Psychiatric diseases: The feeling of anxiety/tension, anxiety disorder/anxiety state, sleep disorders, depressive feeling/sensation, depressive disorders, adaptative response, personality disorders, phobia/compulsive disorder, affective psychosis, schizophrenia, other unspecified psychosis, and anorexia nervosa/bulimia.

### 2.3. Data Analysis

We segment our sample into different geographical regions, taking the five different municipalities sizes (less than 10,000 people, between 10,000 and 49,999, between 49,999 and 99,999, between 100,000 and 499,999, and more than 500.000 people) in each of the six Spanish regions considered in the database. We obtain 25 geographical groups since some regions do not have municipalities of all sizes. The groups have 24,511 patients on average in a range that goes from 4371 to 89,079.

Firstly, the incidence of every disease for every population group was calculated. We excluded incidences lower than 0.1% in our sample for not being representative. Such was the case for dementia, feminine infertility, disability associated with gestation, disability associated with genital apparatus and mammary disease, phobia/compulsive disorder and adaptative response, tuberculosis, leukemia, auditory system cancer, pulmonary embolism, Parkinson’s disease, respiratory system cancer, renal cancer, and urinary bladder cancer.

Secondly, to measure clinical variation, the coefficient of variation among geographical clusters was calculated for disease, which is defined as the ratio of the standard deviation between the geographical groups to the mean (CV = σ/μ).

Thirdly, we study if there are significant differences between the clinical variation in diseases with established protocols diagnosed by specialists, women’s diseases, and psychiatric diseases concerning the control of generic diseases with established protocol groups. The tests are performed using multivariate linear regression models whose target is the natural logarithm of the diagnostic variability of each disease. We realized three estimates. In Estimate 1 we built a multivariate linear regression model of the CV of the three groups of diseases vs. the control group. The target of the model is the CV of each disease, and the covariates are dummy variables indicating whether the disease belongs to each of the four groups we considered. In Estimate 2 we repeat the model in Estimate 1, taking the incidence effect of every disease into account as an additional covariate (as the logarithm of the mean incidence of each disease). This parameter adjusts the fact that diseases with lower prevalence are expected to have higher variability due to the mathematical definition of variability (CV = σ/μ). Recall that we are restricted to diseases with a prevalence of at least 0,1%.

Given that geographical groups have an average of 24,511 patients, assuming that the number of diagnosed patients follows a Poisson distribution, for diseases with 0.1% prevalence, the observed CV is expected to be 0.20 just because of sample size. For diseases with a prevalence of 0.3%, it would be 0.12, while for a prevalence of 1.0%, it would be 0.06. Hence, the variability that comes from a low prevalence vanishes quite fast due to the relatively large sample size of geographical groups, and therefore this effect is not expected to be critical. Indeed, if we consider the 10 diseases with the lowest prevalence, its CV mean is 0.55, while the CV mean for the highest 10 disease prevalence is 0.48. In Estimate 3 we include exogenous and healthcare system effects.

Namely, these include age and income, as exogenous effects, and municipality size to proxy availability of large hospitals as well as other potential exogenous effects, such as lifestyle differences between urban and rural areas. To include the aforementioned effects, we run a two-step regression. In the first step, we built 38 multivariate linear regressions, one per disease, where we adjust whether the patient is diagnosed with the disease using the exogenous factors as covariates. This regression is run at a patient level. In the second step, we use the coefficients of the first step to re-adjust the prevalence of each disease at each geographical region by adding or decreasing it according to the amount attributable to the deviation of the exogenous factors in the region, concerning the average. For instance, if patients in one region were 2 years older than the average, and each year was associated with 0.01 additional prevalence of a certain disease, the adjustment of that region would reduce by 0.02 of its prevalence. The adjustment is performed at a regional level since prevalence is a population metric, not an individual one, and our study is at a population level. Once prevalence is adjusted by exogenous factors, we calculate again the coefficient of variation and run again the same test as in Estimate 2, but with the adjusted variability.

All regressions were run using R.

## 3. Results

### 3.1. Characteristics of the Study Population

The database consists of 617,222 women aged from 15 to 65 with a mean of 43 years, from six regions in Spain: Castilla y León (26% of the sample), Valencia (35%), Andalucía (26%), Canary Islands (13%), Basque Country (11%), and La Rioja (4%).

More basal patient data and disease information are in Table 1 and Table 2.

### 3.2. Calculation of Coefficients of Variation

The coefficients of variation were calculated for every disease (Table 3). The mean coefficient of variation of all diseases was 58.31%. The coefficient of variation for feelings of anxiety/tension is a clear outlier, with a value of 194.4%. Thus, we addressed these items specifically in the robustness check section.

### 3.3. Multivariate Linear Regression Models

The results of the three Estimates described in the methods are presented in Table 4. As can be seen, there is no significant difference in the coefficients of variation between generic and specialty diseases, even when adjusted by prevalence and exogenous factors. Therefore, there is no empirical evidence supporting that the need for a specialist (which can be interpreted both in terms of the availability of better medical resources and as a proxy of diagnostic complexity) increases clinical variation. In all estimates women’s diseases and psychiatric diseases exhibit much larger variability than the other groups, and results are all statistically significant (<0.05). For example, the difference in clinical variability between the control group and mental diseases is 0.3757 (Estimate 1), compared to a base variability for control of 0.3773. When adjusting by prevalence, socio-demographics, and hospital size, this difference reduces to 0.2954, but is still large and statistically significant.

## 4. Discussion

To study the effect of cultural bias in clinical variation, we analyzed two groups of diseases (women’s and psychiatric diseases) with widely known associated bias and taboos [11,16,26,35].

Our results indicate a statistically significantly higher degree of clinical variation in women’s and psychiatric diseases compared with the primary care disease control group. Although no direct causal link can be established between these findings and cognitive bias due to the limitations of our study, the results show strong correlation between them. Using the available data, we systematically ruled out alternative explanations for the observed variation between groups of diseases. Specifically, we accounted for the following: (i) the prevalence effect inherent to the calculation of the coefficient of variation; (ii) variation related to more specialized diseases, which may arise from the need for specialized healthcare resources and knowledge; and (iii) exogenous influences such as age, income, and municipality size, which serve as proxies for hospital availability, population distribution, and lifestyle differences across geographic regions.

After the adjustments, the higher clinical variation in women’s and psychiatric diseases remains statistically significant. We acknowledge that in our study a direct causal relationship between cultural bias and unexplained clinical variability cannot be established. However, the large and statistically significant difference in clinical variation in these two groups of illnesses, which are historically linked to cultural biases, and the absence of alternative explanations for the results, strongly suggest that a relevant and probable causal explanation for the observed results is the existence of cultural biases. Specifically, the estimates show that between 0.2 and 0.3 of the total clinical variation observed in the women’s and psychiatric groups could be explained, among others, by cultural bias.

Consequently, since the average coefficient of variation in women’s and psychiatric diseases is around 0.6 to 0.8, more than one-third of the clinical variation falls in the category of unwarranted clinical variation, which represents a loss in healthcare service quality for those patients [36,37,38,39]. Among the causes of unwarranted variation, inequitable access to resources, poor communication, role confusion, and not applying the best scientific evidence or formation have been defined, with the last one proposed to be the most important. As a measure to improve the stated situation, the authors of this paper agree that the application of formation and standardized protocols based on the best scientific evidence [40,41] could be the best way to reduce cognitive bias associated with cultural taboos, gender bias, and social norms.

Although there are many studies in the literature studying unwarranted variation in specific treatments and medical situations [42,43,44], we have not been able to find any study addressing mental illness taboos and gender bias from this perspective. Particularly, we have been able to find a Spanish study implementing the Atlas of Variations in Medical Practice in the Spanish National Health System, which tackles systematically many pathologies from the perspective of variation, namely orthopedic and trauma surgery, general surgery, pediatric care, diabetes care, etc. [45,46]. Unfortunately for gynecologic pathologies and psychiatric pathologies, the study of these pathologies is critical, since biases may also affect the administration of justice. For example, recent evidence has shown that evaluator-related biases, such as diagnostic-driven and gender-related distortions, may significantly influence forensic psychiatric judgments on criminal responsibility and social dangerousness [47].

As current limitations of our study, data from only 6 of the 17 regions in Spain was available. We lack other exogenous factors that may explain part of the variability at a patient level, such as regular sports practice or social bonds. However, it is difficult to believe that there were significant variations in our groups of around 25,000 patients. Furthermore, male patients were not included in our database, which would constitute an ideal group to compare the variation between males and women in generic diseases with established protocols. Finally, our study relies on a 2017 database with cumulative diagnoses from earlier years; more recent cumulative data (e.g., 2021–2022) are unavailable. However, this limitation is minor, as our focus is on regional variability unexplained by system-related factors and potentially attributable to cultural biases. For example, although COVID-19 may have influenced healthcare system dynamics, it is unlikely to affect cultural biases, and its impact would primarily concern new diagnoses. For further research, analyzing clinical variation in men’s disease, or differences in drug prescription between male and female patients with the same disease could increase the amount of evidence in cultural bias and clinical variation. Future data will also allow assessment of whether unexplained variability has increased or decreased, indicating possible shifts in cultural bias.

## 5. Conclusions

This study finds a statistically significantly higher clinical variation in these disease groups compared to a control group of primary care diseases. While no direct causal association to cultural bias is established, other potential causes of clinical variation differences, such as prevalence effects, specialized diseases, and exogenous factors like age and income, are systematically analyzed and accounted for. After adjustments, the higher clinical variation in women’s and psychiatric diseases remains statistically significant, suggesting that cultural bias may be a significant underlying root cause of these differences. The study estimates that approximately 20–30% of the observed clinical variation in these groups can be attributed to cultural bias. This unwarranted clinical variation could represent a loss in healthcare service quality and even extend its effects beyond clinical outcomes, also influencing medico-legal and forensic judgments [47]. As health systems increasingly adopt AI-based decision-support tools, there is a concrete risk that pre-existing biases will be embedded into algorithmic models, thereby reproducing, or even amplifying the inequities described.

The application of standardized protocols based on scientific evidence, auditing systems, or checks for algorithmic fairness could help reduce cultural bias associated with cultural taboos, gender bias, and social norms. The study acknowledges the lack of research addressing mental illness taboos and gender bias from this perspective and suggests future research on clinical variation in men’s diseases and differences in drug prescription between male and female patients.

## Figures and Tables

**Table 1:** Diseases and prevalence.

Group	Disease	Prevalence
Generic diseases with established protocols	dyslipidemia	17.66%
	Arterial hypertension	11.66%
	Tobacco abuse	8.72%
	Asma	8.20%
	Obesity	5.76%
	Diabetes	3.83%
	Ischemic cardiopathy	0.49%
	Bronchitis	0.33%
	Heart Failure	0.18%
Exclusive women’s diseases	Disability associated with menstruation	12.23%
	Vulvovaginitis	6.87%
	Menopause	4.27%
	Contraception interventions	4.47%
	Fibromyalgia	3.11%
	Uterine Fibroma	2.52%
	Fibrocystic Mastopathy	2.02%
	Endometriosis	0.86%
	Inflammatory pelvic disease	0.20%
Psychiatric diseases	Anxiety disorder	17.36%
	Sleep disorders	5.25%
	Depressive disorders	5.03%
	The feeling of anxiety/tension	1.26%
	Personality disorders	0.87%
	Affective psychoses	0.48%
	Schizophrenia	0.39%
	Anorexia nervosa/bulimia	0.33%
	Other unspecified psychoses	0.25%
Specialist diseases	Lymphoma	0.35%
	Retinal detachment	0.23%
	Macular degeneration	0.13%
	Otosclerosis	0.11%
	Cardiac valvulopathy	0.39%
	Stroke	0.32%
	Rheumatoid Arthritis	0.70%
	Multiple Sclerosis	0.21%
	Epilepsy	0.86%
	Psoriasis	2.03%
	Thyroid neoplasm	0.16%

Note: Dementia, Feminine infertility, Disability associated with gestation, Disability associated with genital apparatus and mammary disease, Phobia/compulsive disorder, Adaptative response, Tuberculosis, Leukemia, Auditory system cancer, Pulmonary embolism, Parkinson’s disease, Respiratory system cancer, Renal cancer, and Urinary bladder cancer were not included in the model for not being representative in our sample (prevalence < 0.01%).

**Table 2:** Socio-demographic data.

		Mean
Age	43.8	
Origin	Castilla y León	26%
	Valencia	35%
	Andalucía	26%
	Canary Islands	13%
	Basque Country	11%
	La Rioja	4%
Municipality size	<10,000 people	15%
	10,000–49,999 people	36%
	50–99,999 people	12%
	100–499,999 people	27%
	≥500,000 people	9%
Income Level	>100,000 E/year	0.33%
	18,000–100,000 E/year	23.27%
	<18,000 E/year	75.33%

**Table 3:** Coefficients of variation of the diseases.

Group	Disease	Coefficient of Variation
Generic diseases with established protocols	Bronchitis	86.8%
	Tobacco abuse	45.1%
	Asma	39.7%
	Heart Failure	34.3%
	Ischemic cardiopathy	30.8%
	Dyslipidemia	30.3%
	Diabetes	30.0%
	Obesity	23.4%
	Arterial hypertension	19.0%
Exclusive women’s diseases	Inflammatory pelvic disease	107.6%
	Contraception interventions	82.8%
	Vulvovaginitis	61.4%
	Uterine Fibroma	59.1%
	Menopause	56.7%
	Fibrocystic Mastopathy	53.9%
	Disability associated with menstruation	49.5%
	Endometriosis	31.3%
Psychiatric diseases	The feeling of anxiety/tension	194.4%
	Sleep disorders	71.2%
	Other unspecified psychoses	65.5%
	Personality disorders	65.1%
	Anxiety disorder	64.4%
	Depressive disorders	63.5%
	Anorexia nervosa/bulimia	48.4%
	Affective psychoses	48.1%
	Schizophrenia	30.2%

**Table 4:** Estimates of the differences between the coefficients of variation of the four groups. *p*-values in brackets.

Disease Type	Coefficient of Variation	Estimate 1	Estimate 2	Estimate 3
Control	0.3773			
Specialty	0.4271	0.0498	0.0299	0.0267
		(0.6820)	(0.9668)	(0.9958)
Women	0.6118	0.2345	0.2364	0.2358
		(0.0225)	(0.0267)	(0.0237)
Mental	0.7530	0.3757	0.3146	0.2954
		(0.0064)	(0.0092)	(0.0132)
Estimate 1: Mean *t*-test. Estimate 2: Adjusted by prevalence. Estimate 3: Adjusted by prevalence, socio-demographics, and hospital size.				

Note: As a robustness check we repeated the stated models taking the outlier value of The feeling of anxiety/tension (CV = 194.4%). The statistical significance remains through all estimates.

## Data Availability

The original contributions presented in this study are included in the article. Further inquiries can be directed to the corresponding author.
